# Epidermoid cyst in tongue's ventral face

**DOI:** 10.1016/S1808-8694(15)30587-5

**Published:** 2015-10-19

**Authors:** Jozinete Vieira Pereira, Pollianna Muniz Alves, Cristina Ruan Ferreira de Araújo, Karuza Maria Alves Pereira, Antônio de Lisboa Lopes Costa

**Affiliations:** 1 PhD. Professor of Stomatology - Universidade Estadual da Paraíba.; 2 MSc. PhD student in oral pathology- UFRN.; 3 MSc. PhD student in oral pathology- UFRN..; 4 MSc. PhD student in oral pathology- UFRN..; 5 PhD. Professor - Graduate Program in Oral Pathology - UFRN. Graduate Program in Oral pathology - Universidade Federal do Rio Grande do Norte (UFRN).

**Keywords:** dermoid cyst, epidermoid cyst, tongue

## INTRODUCTION

Epidermoid cysts are benign development alterations without the presence of adjacent structures such as sebaceous glands, hair follicles or sweat glands. It may start at any part of the body and they are more commonly found in the testicles and ovaries^1^, ^2^. They are rare in the orofacial region, and only about 1% involves the oral cavity^3^. Their etiology is uncertain; however, it is believed that they are associated with ectoderm remains trapped in the first and second branchial arches; even then, there are other theories in vogue such as accidental or surgical events, when epithelium is traumatically implanted within deeper structures^4^. We hereby report a case of epidermoid cyst in the tongue belly, and we discuss the importance of knowledge and the clinical and histological differential diagnosis of this entity.

## CASE REPORT

Female, Caucasian, 60 years of age, complained of a bulging in the belly of her tongue, which she had noticed for about 4 months now. In her mouth we noticed a white-yellowish, smooth, soft, asymptomatic, exophytic, sessile lesion, without trauma history, measuring about 0.5x0.5cm, and the clinical diagnosis was of fibroma or lipoma.

Her routine blood tests were all normal, then we took her to surgery in order to remove the lesion under local anesthesia and the specimen was sent to pathology.

**Figure 1:**
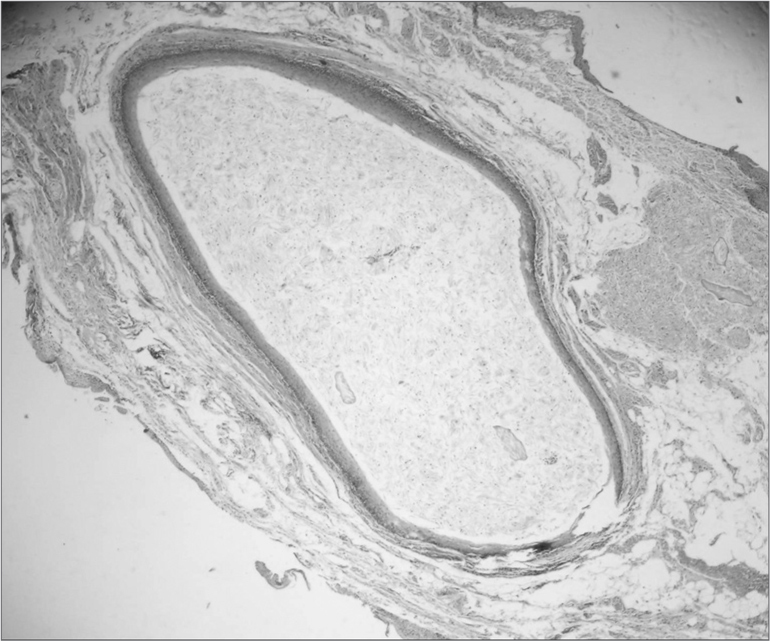
Disorder cavity coated by orthokeratinized stratified transitional epithelium, showing abundant keratin in the cyst lumen (Hematoxylin/Eosin, 100x).

Macroscopically it was a fragment of soft, white-brownish tissue, with rough shape and surface, fibrous, measuring 0.8x0.4x0.5cm. When the specimen was cut, there was oozing of a caseous material from inside. Histopathology we observed a fragment of a cystic cavity, coated by stratified squamous epithelium, with a lumen fully filled by orthokeratin and a capsule made up of dense fibrous connective tissue, with moderate mononuclear inflammatory infiltrate and engorged vessels, and not other skin adjacent structures, thus leading us to the diagnosis of epidermoid cyst (Figure 1).

## DISCUSSION

Epidermoid cysts’ etiology is highly challenged in the literature^3^, ^5^.

Although the dermoid cyst represents a distinct entity, the word dermoid is normally used to identify three different types of cysts: epidermoid (without derm adjacent structures in its coating epithelium), dermoid (with skin adjacent structures, such as sweat glands and hair follicles) and teratoid (coating with structures from the three germinative layers) ^1^. The case hereby presented is an example of an epidermoid cyst, the pathology exam reported a cystic cavity coated by epithelium with orthokeratin inside, without the presence of other skin adjacent structures.

There are few reports of epidermoid cysts on the tongue, especially on its belly, because when it occurs in the oral cavity, it usually happens in the submental region. Therefore, we believe it is important to present this case, adding it to the few reports we have in the literature.

Depending on its location, the differential diagnosis of epidermoid cysts can be made based on infectious processes, ranula, thyroglossal duct cyst, cystic hygroma and fat tissue build up1,^2^, ^4^. As in the case aforementioned, the lesion was on the tongue belly, and according to its clinical characteristics, fibroma or lipoma was suspected.

Cyst location is a determining factor for surgery, suggesting an intraoral approach for the sublingual cyst and a submental approach for the submental and submandibular cysts6, in our case here, it was a small cyst located on the tongue and the patient did not have any abnormality in her lab work up, that is why we chose an intraoral approach.

## FINAL REMARKS

The epidermoid cyst, although rare and benign should not be underestimated. It is important to make the clinical and pathological differential diagnosis. Therefore, it is very important that dentists be aware of this disorder.
